# Breaking barriers in pathology: bridging gaps in multidisciplinary collaboration

**DOI:** 10.1007/s00428-024-03991-6

**Published:** 2024-12-05

**Authors:** Rui Almeida, Ceren Boyaci, Marcin Braun, Federica Pezzuto, Philipp Zens, João Lobo, Dina Tiniakos

**Affiliations:** 1 Serviço de Anatomia Patológica, Unidade Local de Saúde Coimbra, Centro Hospitalar Universitário de Coimbra, Coimbra, Portugal; 2 Department of Oncology-Pathology, Karolinska Institutet, Stockholm, Sweden; 3 Department of Clinical Pathology and Cancer Diagnostics, Karolinska University Hospital, Stockholm, Sweden; 4 Department of Pathology, Chair of Oncology, Medical University of Łódź, Łódź, Poland; 5 Department of Cardiac, Thoracic, Vascular Sciences and Public Health, University of Padova, Padua, Italy; 6 Graduate School for Health Science, University of Bern, Bern, Switzerland; 7 Institute of Tissue Medicine and Pathology, University of Bern, Bern, Switzerland; 8 Department of Pathology, Portuguese Oncology Institute of Porto (IPO Porto)/Porto Comprehensive Cancer Center Raquel Seruca (Porto CCC), Porto, Portugal; 9 Cancer Biology and Epigenetics Group, IPO Porto Research Center (GEBC CI-IPOP), Portuguese Oncology Institute of Porto (IPO Porto), Porto Comprehensive Cancer Center Raquel Seruca (P.CCC) & CI-IPOP@RISE (Health Research Network), Porto, Portugal; 10 Department of Pathology and Molecular Immunology, ICBAS-School of Medicine and Biomedical Sciences, University of Porto, Porto, Portugal; 11 Department of Pathology, Medical School, Aretaieion Hospital, National and Kapodistrian University of Athens, Athens, Greece; 12 Translational and Clinical Research Institute, Faculty of Medical Sciences, Newcastle University, Newcastle Upon Tyne, UK

**Keywords:** Pathology, Multidisciplinary team, Guidelines, Research, Collaboration, Patient management, Diagnosis, Prognosis, Prediction

## Introduction

Pathology is a unique medical specialty, often misperceived as being limited to disease classification based on morphology. However, modern pathologists are central to patient care, collaborating with various medical specialties and bridging basic and clinical research with patient management. They play an integral role in multidisciplinary meetings, contributing to diagnosis, risk stratification, prognosis, biomarker assessment, and clinical trial eligibility [1]. Efficient communication and active involvement in these processes are essential [2].

With the rise of precision medicine, pathologists are increasingly integrating clinical, imaging, macroscopic, histologic, and molecular data. They are pivotal in implementing advanced technologies like digital pathology, artificial intelligence, and bioinformatics for big data analysis (“omics”), liquid biopsies, and clinical performance of circulating biomarkers, among others [3]. Consequently, pathologists must assert their role in multidisciplinary patient management teams, research groups, and clinical guideline development, while actively participating in clinical trials and scientific societies and communicating their practice and contributions in clinical congresses.

**Fig. 1:**
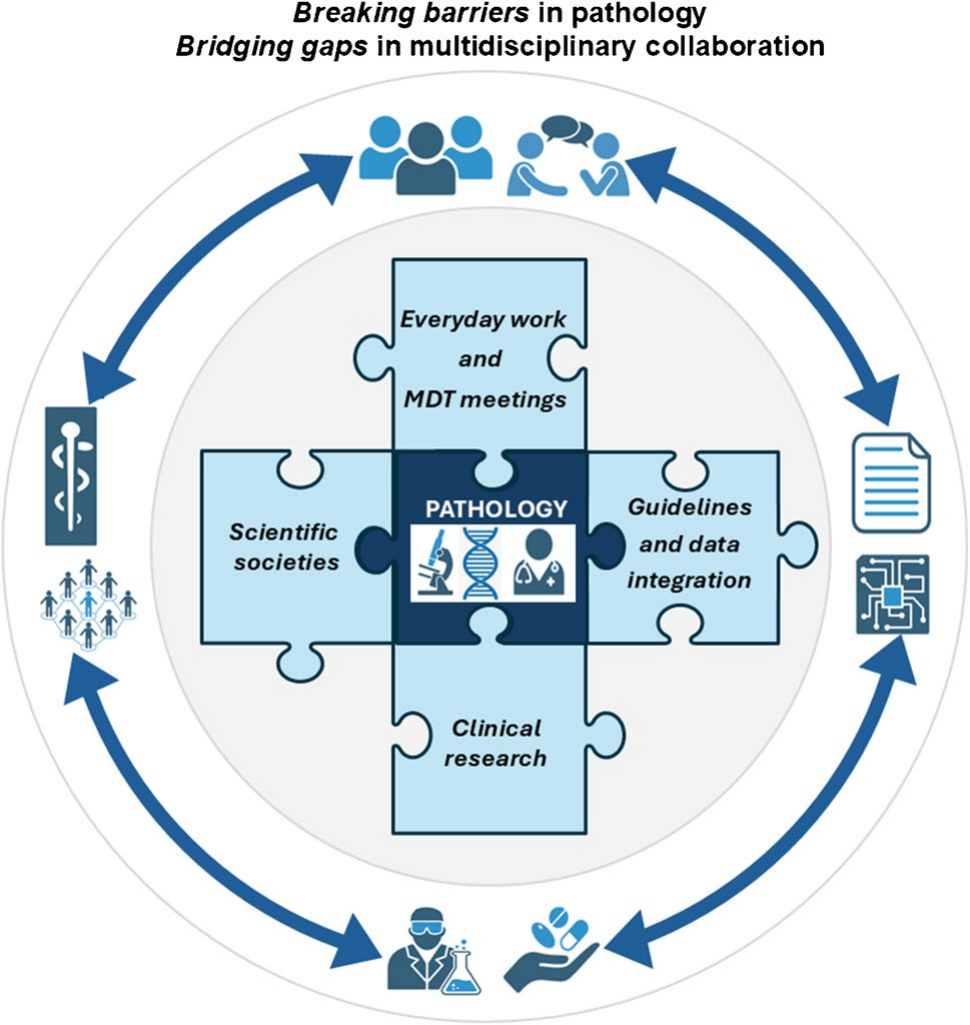
Key areas where pathologists should be actively engaged as members of the professional multidisciplinary team (MDT) optimizing patient care

In this Editorial, we aim to highlight key areas where pathologists should be actively engaged as members of the professional multidisciplinary team (MDT) optimizing patient care (Fig. 1).

## Everyday clinical diagnostic work and multidisciplinary team meetings

A critical aspect of pathology is the daily interaction with clinicians. Effective communication is crucial but is often hampered by limited direct interaction and differences in terminology [2]. To address this, pathologists should participate in case discussions with clinicians, develop standardized reporting templates, and establish clear communication protocols. However, attention to time management and setting boundaries are equally important in order to maintain effective communication without overwhelming working schedules.

In the rapidly evolving field of oncology, regular tumor board meetings are essential for fostering collaboration and integrating diverse expertise in comprehensive treatment plans [4]. Traditionally behind the scenes, pathologists now play more prominent roles in MDT meetings, influencing treatment decisions not only by providing diagnostic, prognostic and predictive information, but also through their deep understanding of disease mechanisms. By actively participating, and even assuming leadership roles, they ensure that diagnostic nuances are fully considered in treatment strategies and that pathology's contributions to patient care are well-integrated [5].

To contribute effectively, pathologists need a thorough understanding of each case, including the patient’s medical history, tumor specifics, and treatment implications. Knowledge of clinical algorithms is vital allowing effective contribution to the decision-making process and aligning their insights with the broader clinical context [6].

## Clinical research

Pathologists are valuable contributors to research, particularly in biomarker studies and precision medicine [7]. They are essential in clinical trial design, where their assessments can determine eligibility for trial entry and/or guide treatment allocations. The evaluation of biomarker status, such as HER2 in breast carcinoma, DNA mismatch repair proteins in endometrial and colorectal carcinoma, and PD-L1 to predict tumor response to immunotherapy, exemplifies their critical role in these processes.

The crucial role of pathologists in clinical trials has been acknowledged in recent guidelines, such as the SPIRIT-Path extension, recommending specific items to guarantee proper documentation and standards for cellular and molecular pathology components [7]. Nevertheless, pathologists’ contributions are often underrecognized, necessitating stronger advocacy for their inclusion as leaders or co-leaders in research projects.

Pathologists involved in research should secure financial independence by pursuing research grants and requiring proper compensation for their contribution in collaborative research grant applications. Their contributions need to be recognized through appropriate authorship in scientific publications, aiming for first or senior author, joint author positions, and/or corresponding author roles when pathology is central to the study. Establishing a leading role in multi-disciplinary research is vital for advancing pathology as a discipline and facilitating its integration into modern research protocols. Therefore, greater advocacy is required to highlight the indispensable role of pathologists in current research practice.

## Guideline development

Clinical practice guidelines are essential for standardizing patient care, especially in diseases that rely on tissue-based diagnostics, such as cancer or autoimmune hepatitis. Pathologists are central for multidisciplinary guideline committees based on their dual role as content experts and as healthcare specialists providing accurate diagnoses and contributing prognostic and predictive information.

Current advances in automation have streamlined most molecular techniques, reducing the need for bioinformatic skills. The focus of molecular pathology is shifting toward the careful selection of appropriate tissue samples and the integration of molecular results with histopathological or cytopathological findings, tasks that can be effectively performed by trained pathologists improving patient-centered management [8]. Thus, their early involvement in guideline development, either as individuals or as members of working groups, allows pathologists’ insights to shape clinical standards from the outset, enhancing patient care effectiveness. Scientific societies, as will be discussed below, play an important role in fostering these collaborations. An example is the effective collaboration of the European Society of Pathology with the European Society of Gynaecological Oncology and the European Society for Radiotherapy and Oncology in developing and updating comprehensive guidelines on the diagnosis and treatment of cervical cancer [9].

## Scientific societies

Scientific societies are fundamental in advancing medicine and improving healthcare quality. They promote professional development by facilitating knowledge exchange and collaboration across geographical regions and disciplines fostering collaboration and sharing [10]. By engaging in clinical scientific societies relevant to their field of expertise, pathologists can promote their daily practices into widely accessible knowledge, enhancing collaboration with other healthcare professionals. Treatment decision meetings, scientific events, and special interest groups provide excellent opportunities for such interactions. Young pathologists should be encouraged to take on leadership roles within these societies, empowering them to influence change, ensuring that their contributions are recognized, and keeping pathology integrated within the broader medical community [11]. Their training and their leading role in molecular pathology, as promoted by pathology scientific societies, are essential prerequisites.

## Conclusion

The dynamic role of pathologists in multidisciplinary teams, basic and clinical research, therapeutic trials, guideline development committees, and scientific societies needs to be emphasized and clarified. There is a need for advocacy within both clinical and pathology disciplines, as well as within industry, to recognize and support the valuable contributions of pathologists to patient care and medical research.
